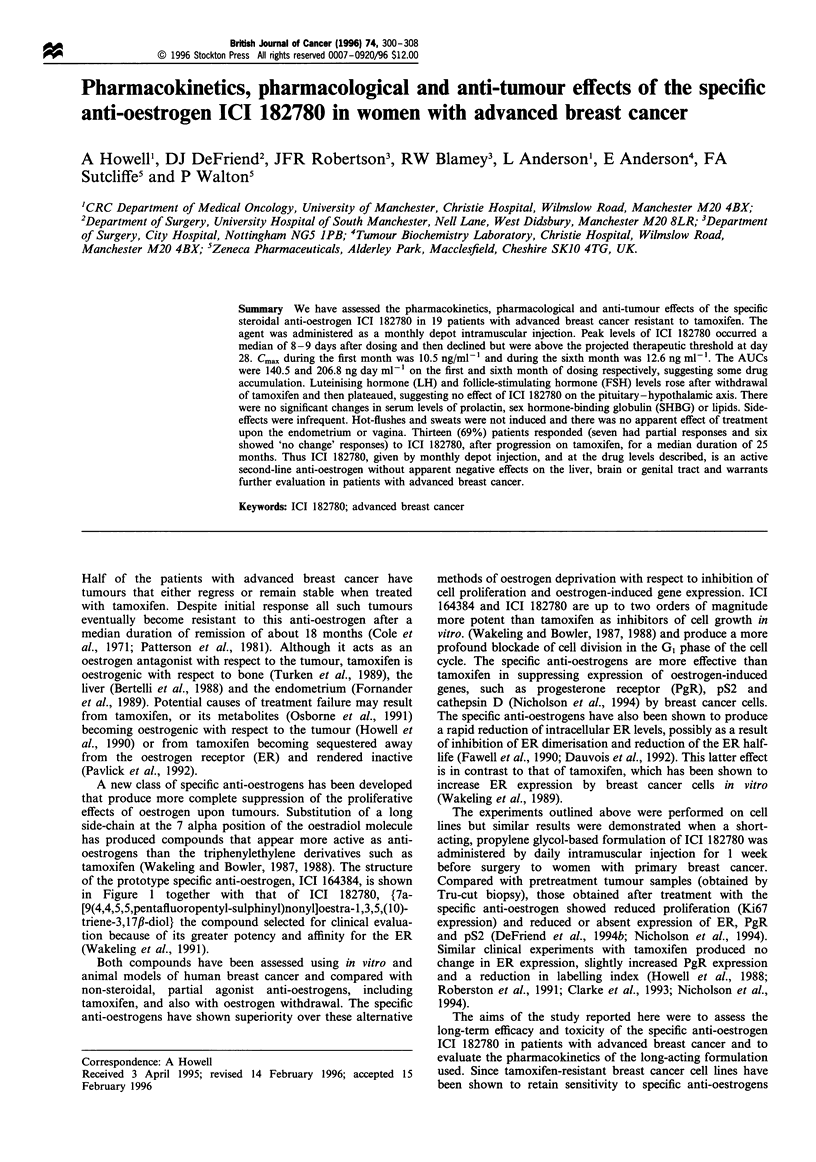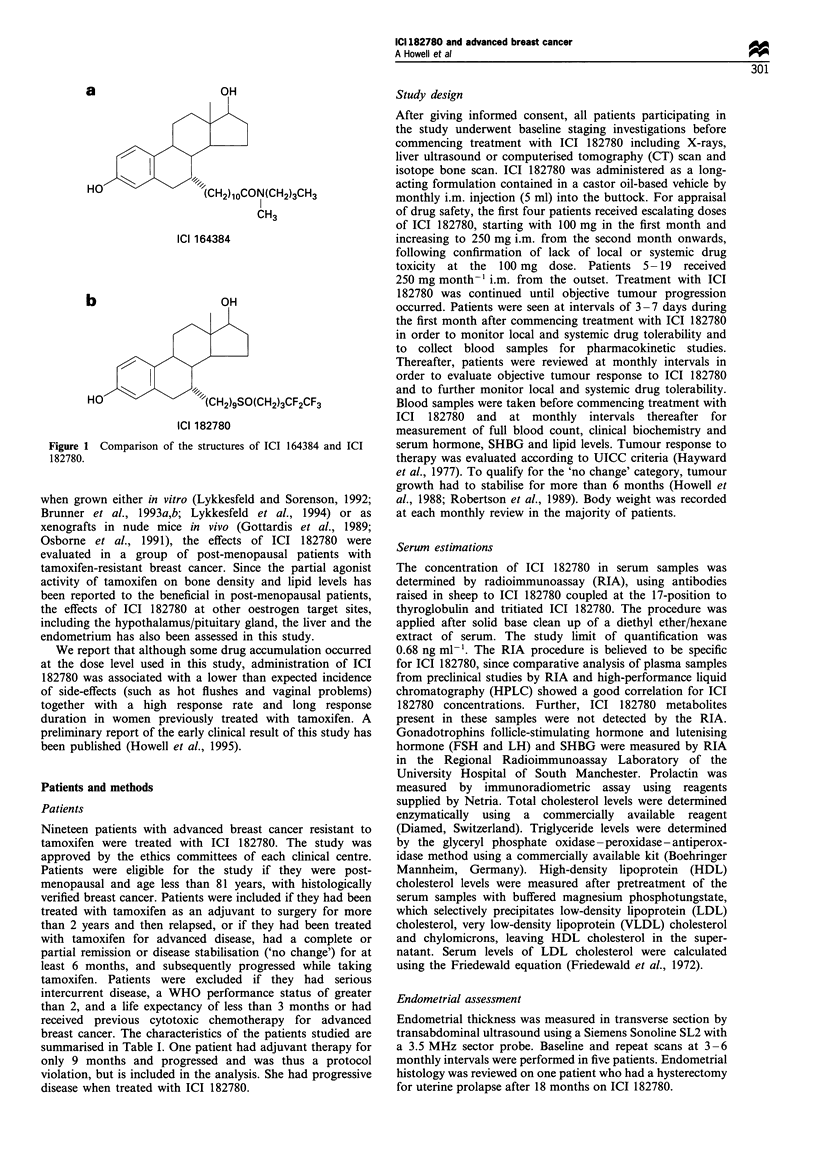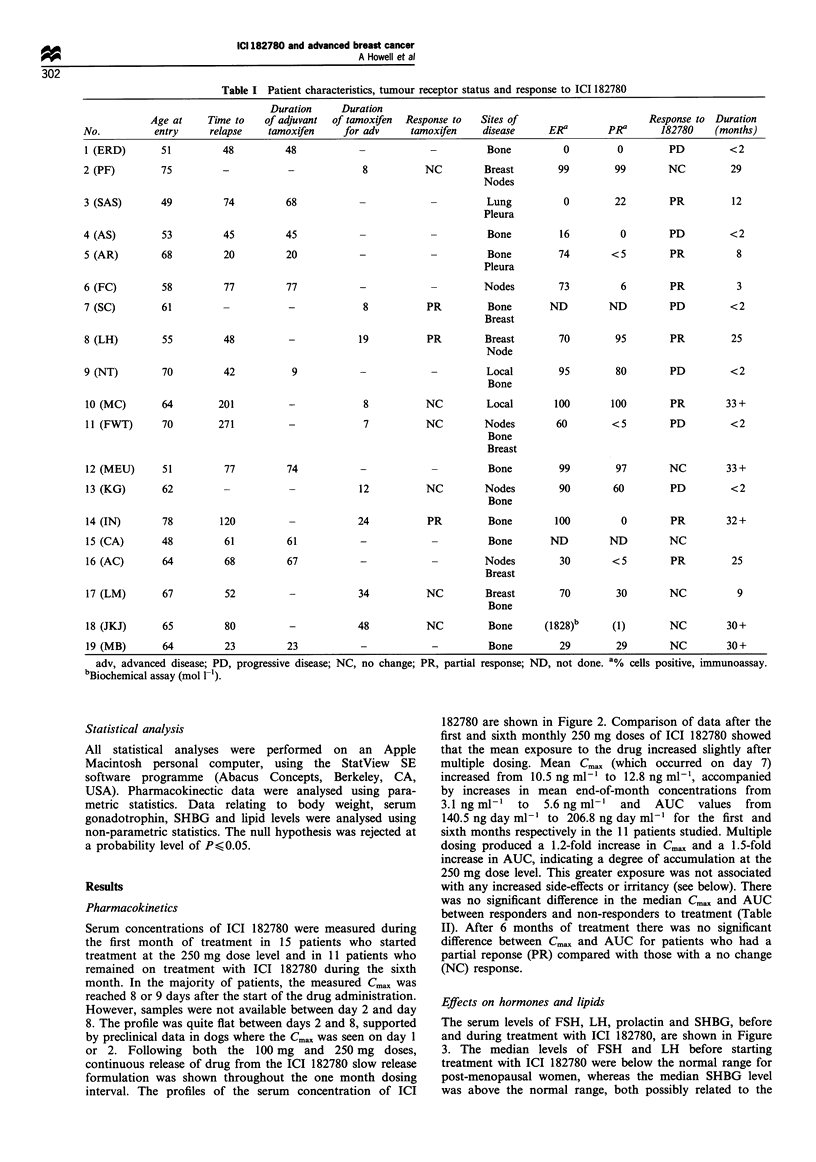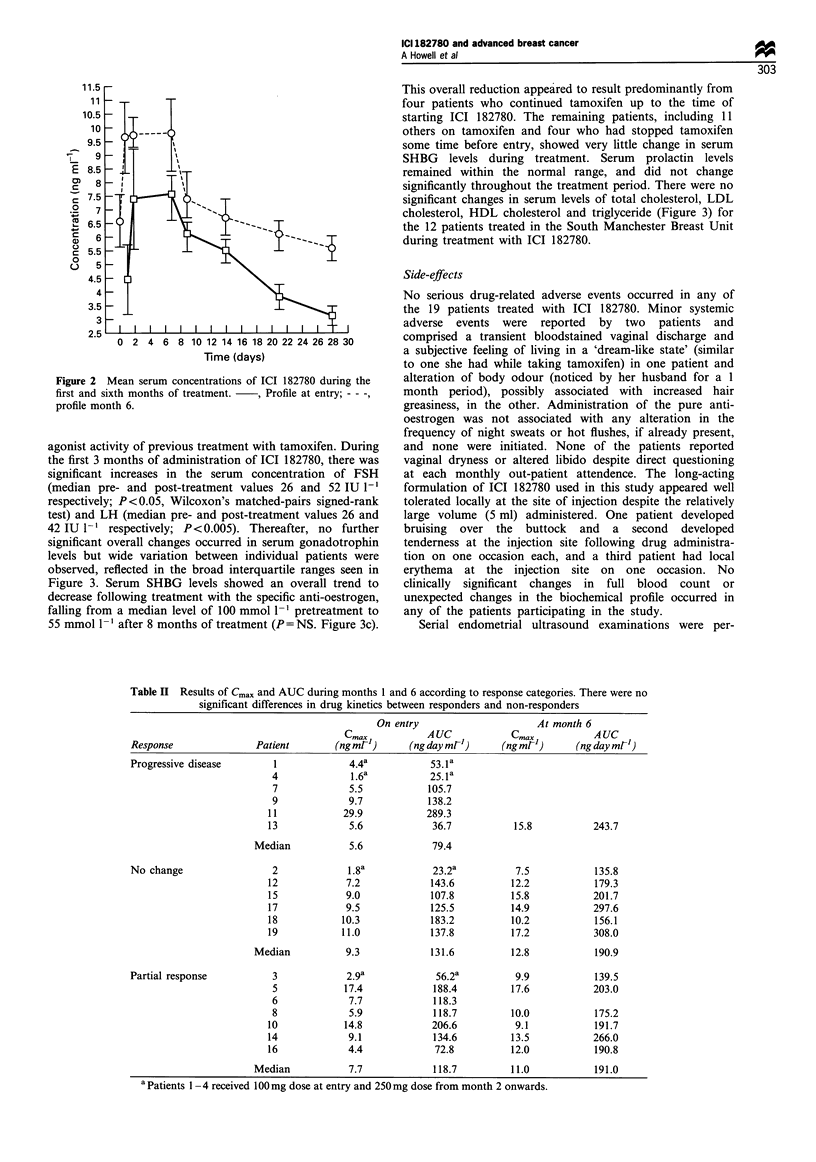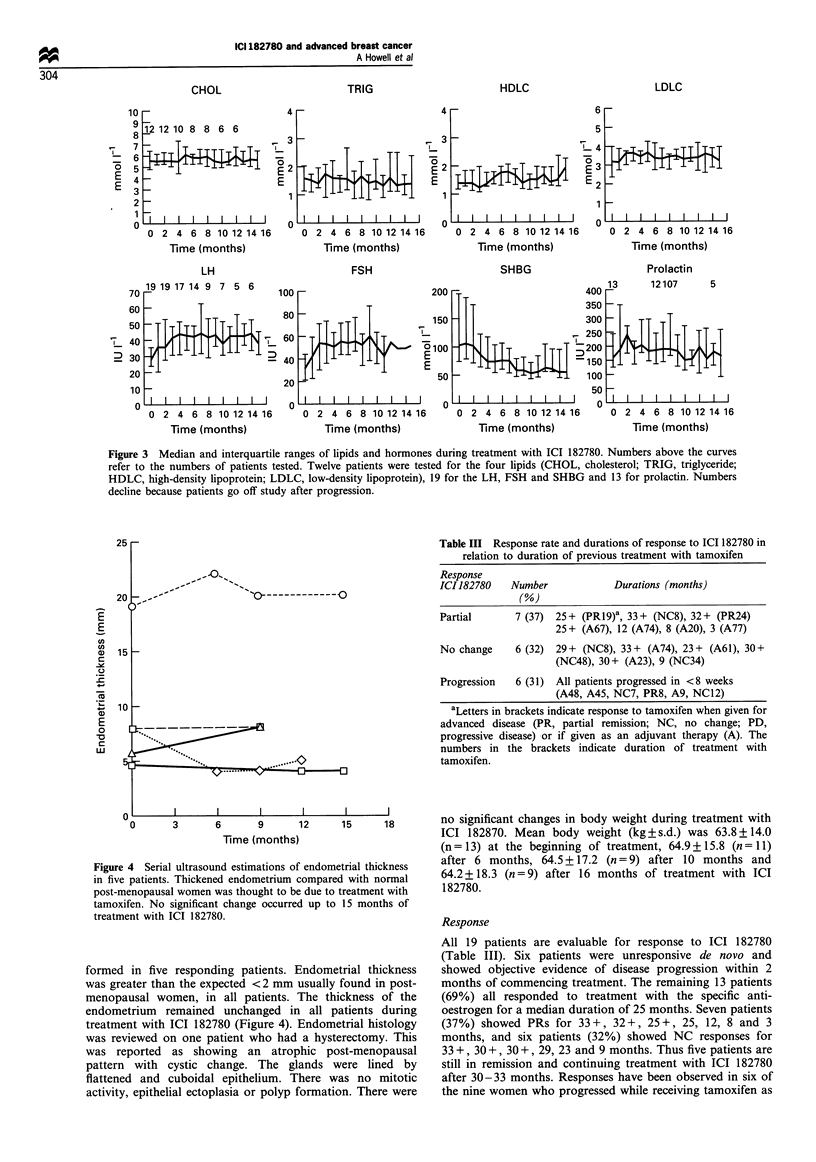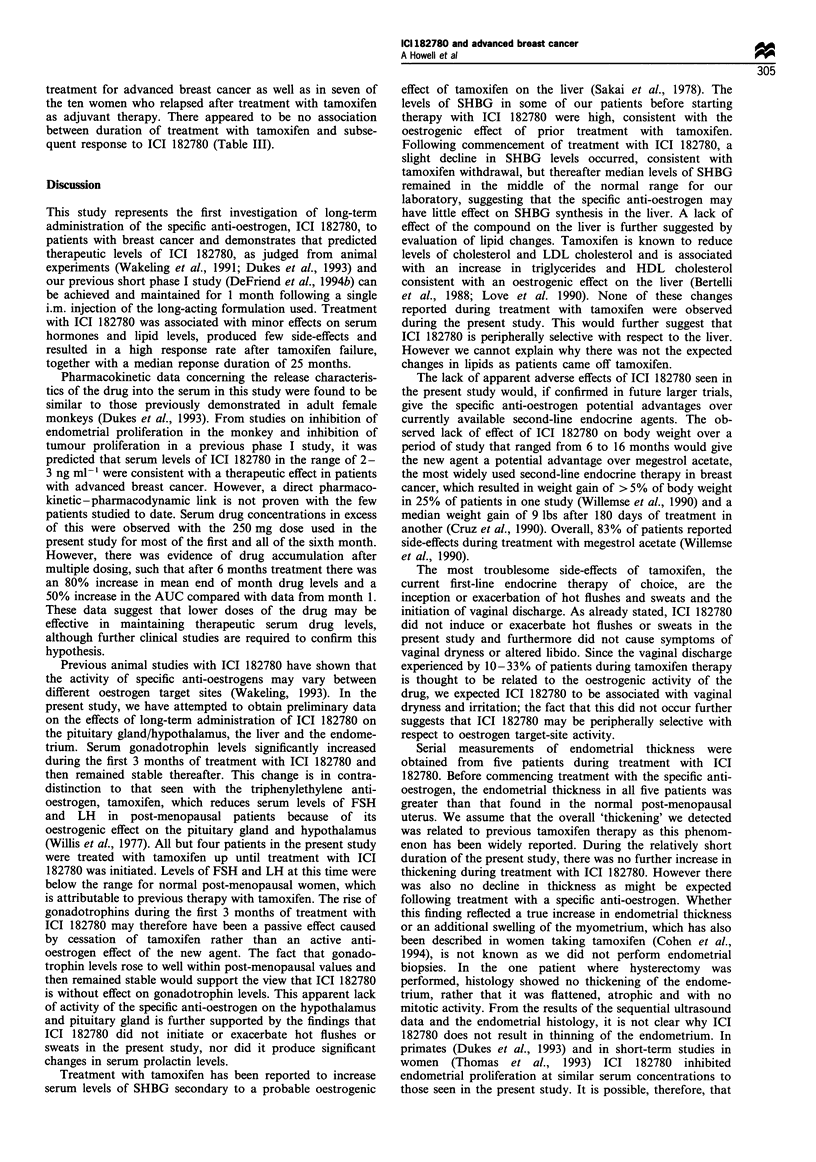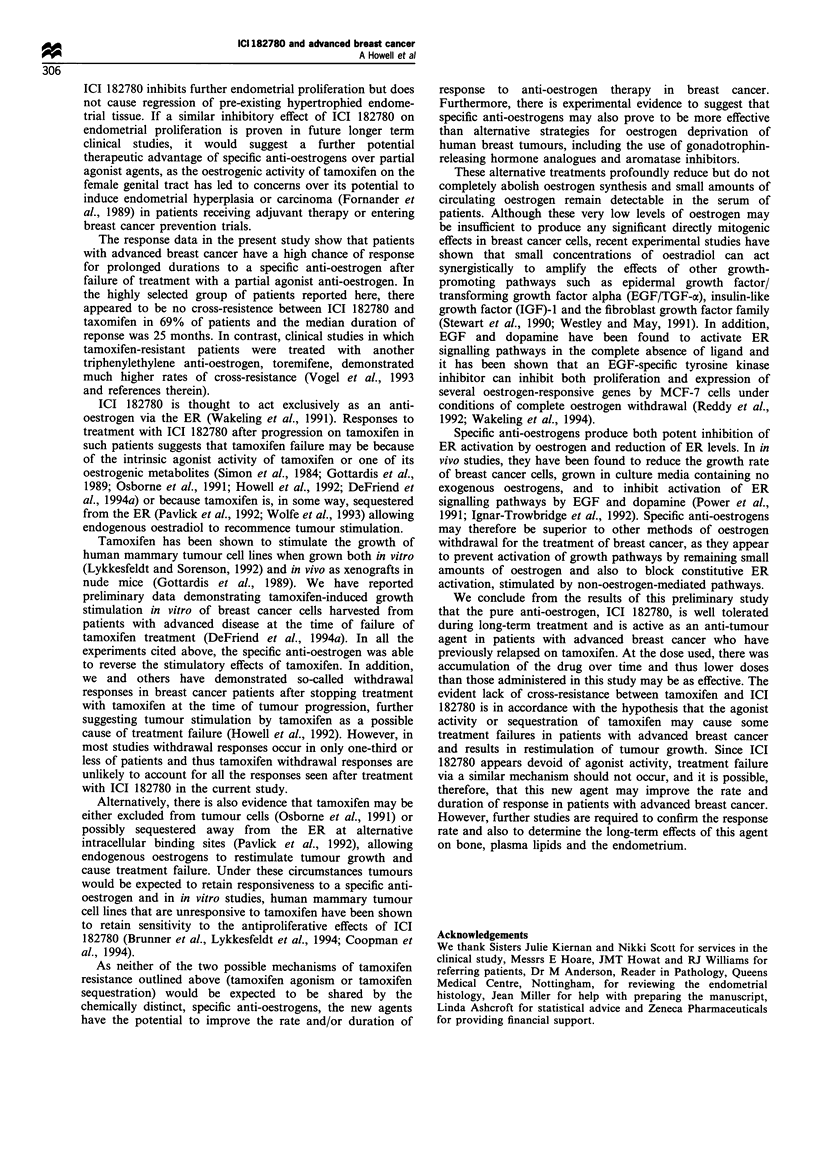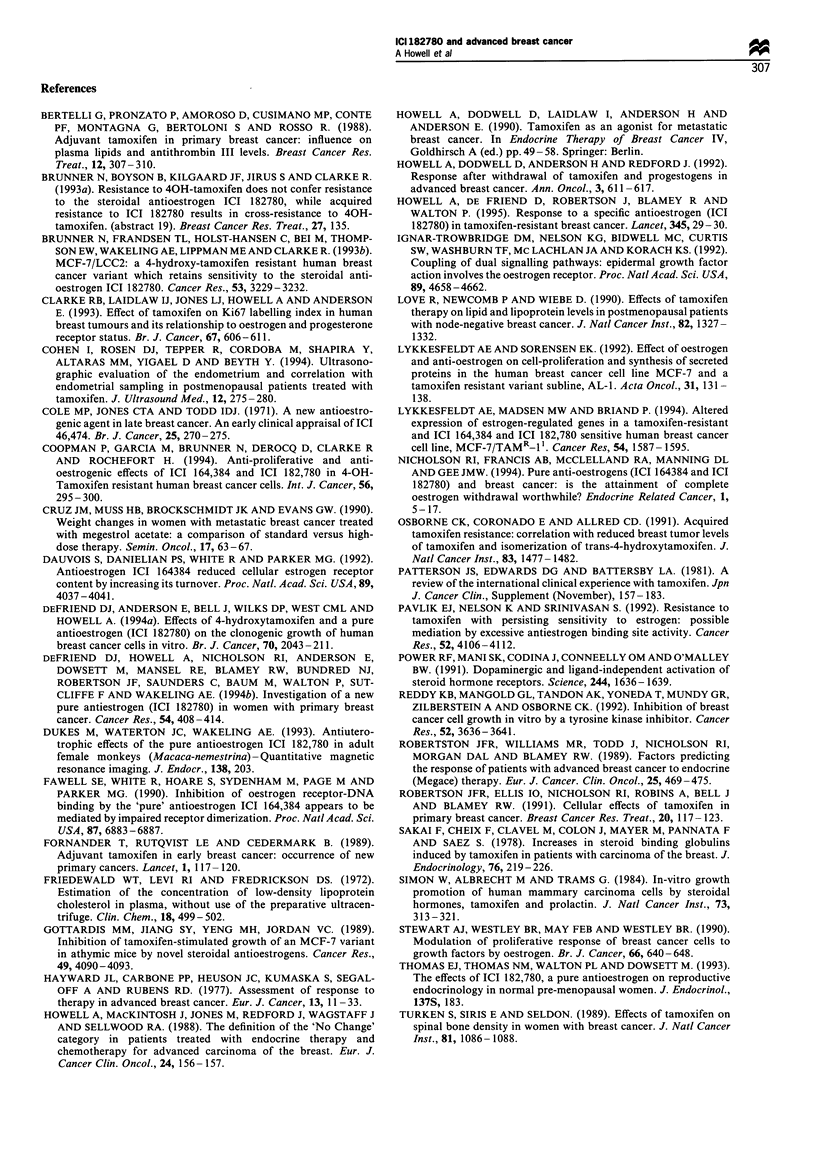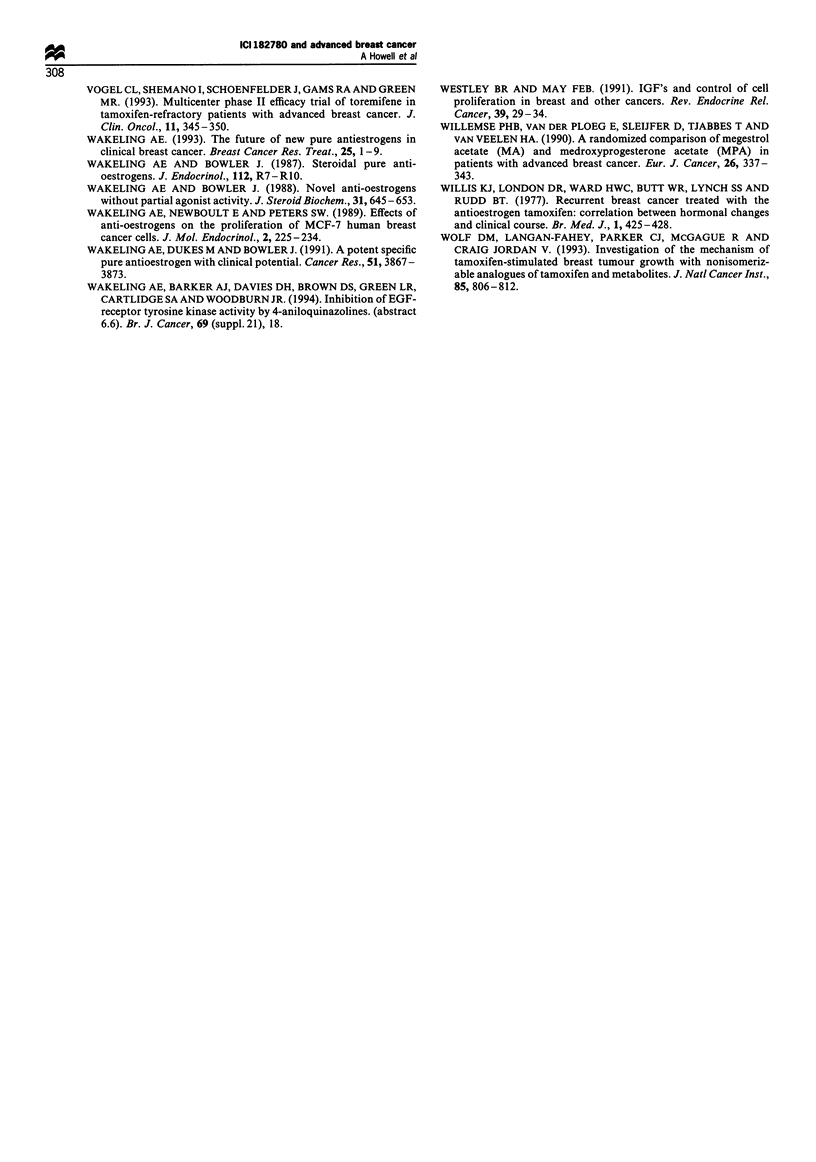# Pharmacokinetics, pharmacological and anti-tumour effects of the specific anti-oestrogen ICI 182780 in women with advanced breast cancer.

**DOI:** 10.1038/bjc.1996.357

**Published:** 1996-07

**Authors:** A. Howell, D. J. DeFriend, J. F. Robertson, R. W. Blamey, L. Anderson, E. Anderson, F. A. Sutcliffe, P. Walton

**Affiliations:** CRC Department of Medical Oncology, University of Manchester, Christie Hospital, UK.

## Abstract

We have assessed the pharmacokinetics, pharmacological and anti-tumour effects of the specific steroidal anti-oestrogen ICI 182780 in 19 patients with advanced breast cancer resistant to tamoxifen. The agent was administered as a monthly depot intramuscular injection. Peak levels of ICI 182780 occurred a median of 8-9 days after dosing and then declined but were above the projected therapeutic threshold at day 28. Cmax during the first month was 10.5 ng/ml-1 and during the sixth month was 12.6 ng ml-1. The AUCs were 140.5 and 206.8 ng day ml-1 on the first and sixth month of dosing respectively, suggesting some drug accumulation. Luteinising hormone (LH) and follicle-stimulating hormone (FSH) levels rose after withdrawal of tamoxifen and then plateaued, suggesting no effect of ICI 182780 on the pituitary-hypothalamic axis. There were no significant changes in serum levels of prolactin, sex hormone-binding globulin (SHBG) or lipids. Side-effects were infrequent. Hot-flushes and sweats were not induced and there was no apparent effect of treatment upon the endometrium or vagina. Thirteen (69%) patients responded (seven had partial responses and six showed "no change' responses) to ICI 182780, after progression on tamoxifen, for a median duration of 25 months. Thus ICI 182780, given by monthly depot injection, and at the drug levels described, is an active second-line anti-oestrogen without apparent negative effects on the liver, brain or genital tract and warrants further evaluation in patients with advanced breast cancer.


British Journal of Cancer (1996) 74, 300-308
© 1996 Stockton Press All rights reserved 0007-0920/96 $12.00

### Pharmacokinetics, pharmacological and anti-tumour effects of the specific anti-oestrogen ICI 182780 in women with advanced breast cancer

A Howell[1], DJ DeFriend[2], JFR Robertson[3], RW Blamey[3], L Anderson[1], E Anderson[4], FA Sutcliffe[5] and P Walton[5]

[1]*CRC Department of Medical Oncology, University of Manchester, Christie Hospital, Wilmslow Road, Manchester M20 4BX;* [2]*Department of Surgery, University Hospital of South Manchester, Nell Lane, West Didsbury, Manchester M20 8LR;* [3]*Department of Surgery, City Hospital, Nottingham NG5 1PB;* [4]*Tumour Biochemistry Laboratory, Christie Hospital, Wilmslow Road, Manchester M20 4BX;* [5]*Zeneca Pharmaceuticals, Alderley Park, Macclesfield, Cheshire SK10 4TG, UK.*

**Summary** We have assessed the pharmacokinetics, pharmacological and anti-tumour effects of the specific steroidal anti-oestrogen ICI 182780 in 19 patients with advanced breast cancer resistant to tamoxifen. The agent was administered as a monthly depot intramuscular injection. Peak levels of ICI 182780 occurred a median of 8–9 days after dosing and then declined but were above the projected therapeutic threshold at day 28. $C_{max}$ during the first month was 10.5 ng/ml$^{-1}$ and during the sixth month was 12.6 ng ml$^{-1}$. The AUCs were 140.5 and 206.8 ng day ml$^{-1}$ on the first and sixth month of dosing respectively, suggesting some drug accumulation. Luteinising hormone (LH) and follicle-stimulating hormone (FSH) levels rose after withdrawal of tamoxifen and then plateaued, suggesting no effect of ICI 182780 on the pituitary–hypothalamic axis. There were no significant changes in serum levels of prolactin, sex hormone-binding globulin (SHBG) or lipids. Side-effects were infrequent. Hot-flushes and sweats were not induced and there was no apparent effect of treatment upon the endometrium or vagina. Thirteen (69%) patients responded (seven had partial responses and six showed 'no change' responses) to ICI 182780, after progression on tamoxifen, for a median duration of 25 months. Thus ICI 182780, given by monthly depot injection, and at the drug levels described, is an active second-line anti-oestrogen without apparent negative effects on the liver, brain or genital tract and warrants further evaluation in patients with advanced breast cancer.

**Keywords:** ICI 182780; advanced breast cancer

Half of the patients with advanced breast cancer have tumours that either regress or remain stable when treated with tamoxifen. Despite initial response all such tumours eventually become resistant to this anti-oestrogen after a median duration of remission of about 18 months (Cole *et al.*, 1971; Patterson *et al.*, 1981). Although it acts as an oestrogen antagonist with respect to the tumour, tamoxifen is oestrogenic with respect to bone (Turken *et al.*, 1989), the liver (Bertelli *et al.*, 1988) and the endometrium (Fornander *et al.*, 1989). Potential causes of treatment failure may result from tamoxifen, or its metabolites (Osborne *et al.*, 1991) becoming oestrogenic with respect to the tumour (Howell *et al.*, 1990) or from tamoxifen becoming sequestered away from the oestrogen receptor (ER) and rendered inactive (Pavlick *et al.*, 1992).

A new class of specific anti-oestrogens has been developed that produce more complete suppression of the proliferative effects of oestrogen upon tumours. Substitution of a long side-chain at the 7 alpha position of the oestradiol molecule has produced compounds that appear more active as anti-oestrogens than the triphenylethylene derivatives such as tamoxifen (Wakeling and Bowler, 1987, 1988). The structure of the prototype specific anti-oestrogen, ICI 164384, is shown in Figure 1 together with that of ICI 182780, {7a-[9(4,4,5,5,pentafluoropentyl-sulphinyl)nonyl]oestra-1,3,5,(10)-triene-3,17$\beta$-diol} the compound selected for clinical evaluation because of its greater potency and affinity for the ER (Wakeling *et al.*, 1991).

Both compounds have been assessed using *in vitro* and animal models of human breast cancer and compared with non-steroidal, partial agonist anti-oestrogens, including tamoxifen, and also with oestrogen withdrawal. The specific anti-oestrogens have shown superiority over these alternative methods of oestrogen deprivation with respect to inhibition of cell proliferation and oestrogen-induced gene expression. ICI 164384 and ICI 182780 are up to two orders of magnitude more potent than tamoxifen as inhibitors of cell growth *in vitro*. (Wakeling and Bowler, 1987, 1988) and produce a more profound blockade of cell division in the $G_1$ phase of the cell cycle. The specific anti-oestrogens are more effective than tamoxifen in suppressing expression of oestrogen-induced genes, such as progesterone receptor (PgR), pS2 and cathepsin D (Nicholson *et al.*, 1994) by breast cancer cells. The specific anti-oestrogens have also been shown to produce a rapid reduction of intracellular ER levels, possibly as a result of inhibition of ER dimerisation and reduction of the ER half-life (Fawell *et al.*, 1990; Dauvois *et al.*, 1992). This latter effect is in contrast to that of tamoxifen, which has been shown to increase ER expression by breast cancer cells *in vitro* (Wakeling *et al.*, 1989).

The experiments outlined above were performed on cell lines but similar results were demonstrated when a short-acting, propylene glycol-based formulation of ICI 182780 was administered by daily intramuscular injection for 1 week before surgery to women with primary breast cancer. Compared with pretreatment tumour samples (obtained by Tru-cut biopsy), those obtained after treatment with the specific anti-oestrogen showed reduced proliferation (Ki67 expression) and reduced or absent expression of ER, PgR and pS2 (DeFriend *et al.*, 1994*b*; Nicholson *et al.*, 1994). Similar clinical experiments with tamoxifen produced no change in ER expression, slightly increased PgR expression and a reduction in labelling index (Howell *et al.*, 1988; Roberston *et al.*, 1991; Clarke *et al.*, 1993; Nicholson *et al.*, 1994).

The aims of the study reported here were to assess the long-term efficacy and toxicity of the specific anti-oestrogen ICI 182780 in patients with advanced breast cancer and to evaluate the pharmacokinetics of the long-acting formulation used. Since tamoxifen-resistant breast cancer cell lines have been shown to retain sensitivity to specific anti-oestrogens

Correspondence: A Howell
Received 3 April 1995; revised 14 February 1996; accepted 15 February 1996

**a**

ICI 164384

**b**

ICI 182780

**Figure 1** Comparison of the structures of ICI 164384 and ICI 182780.

when grown either *in vitro* (Lykkesfeld and Sorenson, 1992; Brunner *et al.*, 1993*a,b*; Lykkesfeld *et al.*, 1994) or as xenografts in nude mice *in vivo* (Gottardis *et al.*, 1989; Osborne *et al.*, 1991), the effects of ICI 182780 were evaluated in a group of post-menopausal patients with tamoxifen-resistant breast cancer. Since the partial agonist activity of tamoxifen on bone density and lipid levels has been reported to the beneficial in post-menopausal patients, the effects of ICI 182780 at other oestrogen target sites, including the hypothalamus/pituitary gland, the liver and the endometrium has also been assessed in this study.

We report that although some drug accumulation occurred at the dose level used in this study, administration of ICI 182780 was associated with a lower than expected incidence of side-effects (such as hot flushes and vaginal problems) together with a high response rate and long response duration in women previously treated with tamoxifen. A preliminary report of the early clinical result of this study has been published (Howell *et al.*, 1995).

### Patients and methods

### *Patients*

Nineteen patients with advanced breast cancer resistant to tamoxifen were treated with ICI 182780. The study was approved by the ethics committees of each clinical centre. Patients were eligible for the study if they were post-menopausal and age less than 81 years, with histologically verified breast cancer. Patients were included if they had been treated with tamoxifen as an adjuvant to surgery for more than 2 years and then relapsed, or if they had been treated with tamoxifen for advanced disease, had a complete or partial remission or disease stabilisation ('no change') for at least 6 months, and subsequently progressed while taking tamoxifen. Patients were excluded if they had serious intercurrent disease, a WHO performance status of greater than 2, and a life expectancy of less than 3 months or had received previous cytotoxic chemotherapy for advanced breast cancer. The characteristics of the patients studied are summarised in Table I. One patient had adjuvant therapy for only 9 months and progressed and was thus a protocol violation, but is included in the analysis. She had progressive disease when treated with ICI 182780.

### *Study design*

After giving informed consent, all patients participating in the study underwent baseline staging investigations before commencing treatment with ICI 182780 including X-rays, liver ultrasound or computerised tomography (CT) scan and isotope bone scan. ICI 182780 was administered as a long-acting formulation contained in a castor oil-based vehicle by monthly i.m. injection (5 ml) into the buttock. For appraisal of drug safety, the first four patients received escalating doses of ICI 182780, starting with 100 mg in the first month and increasing to 250 mg i.m. from the second month onwards, following confirmation of lack of local or systemic drug toxicity at the 100 mg dose. Patients 5–19 received 250 mg month$^{-1}$ i.m. from the outset. Treatment with ICI 182780 was continued until objective tumour progression occurred. Patients were seen at intervals of 3–7 days during the first month after commencing treatment with ICI 182780 in order to monitor local and systemic drug tolerability and to collect blood samples for pharmacokinetic studies. Thereafter, patients were reviewed at monthly intervals in order to evaluate objective tumour response to ICI 182780 and to further monitor local and systemic drug tolerability. Blood samples were taken before commencing treatment with ICI 182780 and at monthly intervals thereafter for measurement of full blood count, clinical biochemistry and serum hormone, SHBG and lipid levels. Tumour response to therapy was evaluated according to UICC criteria (Hayward *et al.*, 1977). To qualify for the 'no change' category, tumour growth had to stabilise for more than 6 months (Howell *et al.*, 1988; Robertson *et al.*, 1989). Body weight was recorded at each monthly review in the majority of patients.

### *Serum estimations*

The concentration of ICI 182780 in serum samples was determined by radioimmunoassay (RIA), using antibodies raised in sheep to ICI 182780 coupled at the 17-position to thyroglobulin and tritiated ICI 182780. The procedure was applied after solid base clean up of a diethyl ether/hexane extract of serum. The study limit of quantification was 0.68 ng ml$^{-1}$. The RIA procedure is believed to be specific for ICI 182780, since comparative analysis of plasma samples from preclinical studies by RIA and high-performance liquid chromatography (HPLC) showed a good correlation for ICI 182780 concentrations. Further, ICI 182780 metabolites present in these samples were not detected by the RIA. Gonadotrophins follicle-stimulating hormone and lutenising hormone (FSH and LH) and SHBG were measured by RIA in the Regional Radioimmunoassay Laboratory of the University Hospital of South Manchester. Prolactin was measured by immunoradiometric assay using reagents supplied by Netria. Total cholesterol levels were determined enzymatically using a commercially available reagent (Diamed, Switzerland). Triglyceride levels were determined by the glyceryl phosphate oxidase–peroxidase–antiperoxidase method using a commercially available kit (Boehringer Mannheim, Germany). High-density lipoprotein (HDL) cholesterol levels were measured after pretreatment of the serum samples with buffered magnesium phosphotungstate, which selectively precipitates low-density lipoprotein (LDL) cholesterol, very low-density lipoprotein (VLDL) cholesterol and chylomicrons, leaving HDL cholesterol in the supernatant. Serum levels of LDL cholesterol were calculated using the Friedewald equation (Friedewald *et al.*, 1972).

### *Endometrial assessment*

Endometrial thickness was measured in transverse section by transabdominal ultrasound using a Siemens Sonoline SL2 with a 3.5 MHz sector probe. Baseline and repeat scans at 3–6 monthly intervals were performed in five patients. Endometrial histology was reviewed on one patient who had a hysterectomy for uterine prolapse after 18 months on ICI 182780.

**Table I** Patient characteristics, tumour receptor status and response to ICI 182780

| *No.* | *Age at entry* | *Time to relapse* | *Duration of adjuvant tamoxifen* | *Duration of tamoxifen for adv* | *Response to tamoxifen* | *Sites of disease* | *ER[a]* | *PR[a]* | *Response to 182780* | *Duration (months)* |
|---|---|---|---|---|---|---|---|---|---|---|
| 1 (ERD) | 51 | 48 | 48 | – | – | Bone | 0 | 0 | PD | <2 |
| 2 (PF) | 75 | – | – | 8 | NC | Breast Nodes | 99 | 99 | NC | 29 |
| 3 (SAS) | 49 | 74 | 68 | – | – | Lung Pleura | 0 | 22 | PR | 12 |
| 4 (AS) | 53 | 45 | 45 | – | – | Bone | 16 | 0 | PD | <2 |
| 5 (AR) | 68 | 20 | 20 | – | – | Bone Pleura | 74 | <5 | PR | 8 |
| 6 (FC) | 58 | 77 | 77 | – | – | Nodes | 73 | 6 | PR | 3 |
| 7 (SC) | 61 | – | – | 8 | PR | Bone Breast | ND | ND | PD | <2 |
| 8 (LH) | 55 | 48 | – | 19 | PR | Breast Node | 70 | 95 | PR | 25 |
| 9 (NT) | 70 | 42 | 9 | – | – | Local Bone | 95 | 80 | PD | <2 |
| 10 (MC) | 64 | 201 | – | 8 | NC | Local | 100 | 100 | PR | 33+ |
| 11 (FWT) | 70 | 271 | – | 7 | NC | Nodes Bone Breast | 60 | <5 | PD | <2 |
| 12 (MEU) | 51 | 77 | 74 | – | – | Bone | 99 | 97 | NC | 33+ |
| 13 (KG) | 62 | – | – | 12 | NC | Nodes Bone | 90 | 60 | PD | <2 |
| 14 (IN) | 78 | 120 | – | 24 | PR | Bone | 100 | 0 | PR | 32+ |
| 15 (CA) | 48 | 61 | 61 | – | – | Bone | ND | ND | NC | |
| 16 (AC) | 64 | 68 | 67 | – | – | Nodes Breast | 30 | <5 | PR | 25 |
| 17 (LM) | 67 | 52 | – | 34 | NC | Breast Bone | 70 | 30 | NC | 9 |
| 18 (JKJ) | 65 | 80 | – | 48 | NC | Bone | (1828)[b] | (1) | NC | 30+ |
| 19 (MB) | 64 | 23 | 23 | – | – | Bone | 29 | 29 | NC | 30+ |

adv, advanced disease; PD, progressive disease; NC, no change; PR, partial response; ND, not done. [a]% cells positive, immunoassay. [b]Biochemical assay (mol $l^{-1}$).

*Statistical analysis*

All statistical analyses were performed on an Apple Macintosh personal computer, using the StatView SE software programme (Abacus Concepts, Berkeley, CA, USA). Pharmacokinectic data were analysed using parametric statistics. Data relating to body weight, serum gonadotrophin, SHBG and lipid levels were analysed using non-parametric statistics. The null hypothesis was rejected at a probability level of $P \leqslant 0.05$.

### Results

*Pharmacokinetics*

Serum concentrations of ICI 182780 were measured during the first month of treatment in 15 patients who started treatment at the 250 mg dose level and in 11 patients who remained on treatment with ICI 182780 during the sixth month. In the majority of patients, the measured $C_{max}$ was reached 8 or 9 days after the start of the drug administration. However, samples were not available between day 2 and day 8. The profile was quite flat between days 2 and 8, supported by preclinical data in dogs where the $C_{max}$ was seen on day 1 or 2. Following both the 100 mg and 250 mg doses, continuous release of drug from the ICI 182780 slow release formulation was shown throughout the one month dosing interval. The profiles of the serum concentration of ICI 182780 are shown in Figure 2. Comparison of data after the first and sixth monthly 250 mg doses of ICI 182780 showed that the mean exposure to the drug increased slightly after multiple dosing. Mean $C_{max}$ (which occurred on day 7) increased from 10.5 ng $ml^{-1}$ to 12.8 ng $ml^{-1}$, accompanied by increases in mean end-of-month concentrations from 3.1 ng $ml^{-1}$ to 5.6 ng $ml^{-1}$ and AUC values from 140.5 ng day $ml^{-1}$ to 206.8 ng day $ml^{-1}$ for the first and sixth months respectively in the 11 patients studied. Multiple dosing produced a 1.2-fold increase in $C_{max}$ and a 1.5-fold increase in AUC, indicating a degree of accumulation at the 250 mg dose level. This greater exposure was not associated with any increased side-effects or irritancy (see below). There was no significant difference in the median $C_{max}$ and AUC between responders and non-responders to treatment (Table II). After 6 months of treatment there was no significant difference between $C_{max}$ and AUC for patients who had a partial reponse (PR) compared with those with a no change (NC) response.

*Effects on hormones and lipids*

The serum levels of FSH, LH, prolactin and SHBG, before and during treatment with ICI 182780, are shown in Figure 3. The median levels of FSH and LH before starting treatment with ICI 182780 were below the normal range for post-menopausal women, whereas the median SHBG level was above the normal range, both possibly related to the

**Figure 2** Mean serum concentrations of ICI 182780 during the first and sixth months of treatment. ——, Profile at entry; - - -, profile month 6.

agonist activity of previous treatment with tamoxifen. During the first 3 months of administration of ICI 182780, there was significant increases in the serum concentration of FSH (median pre- and post-treatment values 26 and 52 IU $l^{-1}$ respectively; $P<0.05$, Wilcoxon's matched-pairs signed-rank test) and LH (median pre- and post-treatment values 26 and 42 IU $l^{-1}$ respectively; $P<0.005$). Thereafter, no further significant overall changes occurred in serum gonadotrophin levels but wide variation between individual patients were observed, reflected in the broad interquartile ranges seen in Figure 3. Serum SHBG levels showed an overall trend to decrease following treatment with the specific anti-oestrogen, falling from a median level of 100 mmol $l^{-1}$ pretreatment to 55 mmol $l^{-1}$ after 8 months of treatment ($P$=NS. Figure 3c). This overall reduction appeared to result predominantly from four patients who continued tamoxifen up to the time of starting ICI 182780. The remaining patients, including 11 others on tamoxifen and four who had stopped tamoxifen some time before entry, showed very little change in serum SHBG levels during treatment. Serum prolactin levels remained within the normal range, and did not change significantly throughout the treatment period. There were no significant changes in serum levels of total cholesterol, LDL cholesterol, HDL cholesterol and triglyceride (Figure 3) for the 12 patients treated in the South Manchester Breast Unit during treatment with ICI 182780.

*Side-effects*

No serious drug-related adverse events occurred in any of the 19 patients treated with ICI 182780. Minor systemic adverse events were reported by two patients and comprised a transient bloodstained vaginal discharge and a subjective feeling of living in a 'dream-like state' (similar to one she had while taking tamoxifen) in one patient and alteration of body odour (noticed by her husband for a 1 month period), possibly associated with increased hair greasiness, in the other. Administration of the pure anti-oestrogen was not associated with any alteration in the frequency of night sweats or hot flushes, if already present, and none were initiated. None of the patients reported vaginal dryness or altered libido despite direct questioning at each monthly out-patient attendence. The long-acting formulation of ICI 182780 used in this study appeared well tolerated locally at the site of injection despite the relatively large volume (5 ml) administered. One patient developed bruising over the buttock and a second developed tenderness at the injection site following drug administration on one occasion each, and a third patient had local erythema at the injection site on one occasion. No clinically significant changes in full blood count or unexpected changes in the biochemical profile occurred in any of the patients participating in the study.

Serial endometrial ultrasound examinations were per-

**Table II** Results of $C_{max}$ and AUC during months 1 and 6 according to response categories. There were no significant differences in drug kinetics between responders and non-responders

| | | *On entry* | | *At month 6* | |
|---|---|---|---|---|---|
| *Response* | *Patient* | $C_{max}$ *(ng ml$^{-1}$)* | *AUC (ng day ml$^{-1}$)* | $C_{max}$ *(ng ml$^{-1}$)* | *AUC (ng day ml$^{-1}$)* |
| Progressive disease | 1 | 4.4[a] | 53.1[a] | | |
| | 4 | 1.6[a] | 25.1[a] | | |
| | 7 | 5.5 | 105.7 | | |
| | 9 | 9.7 | 138.2 | | |
| | 11 | 29.9 | 289.3 | | |
| | 13 | 5.6 | 36.7 | 15.8 | 243.7 |
| | Median | 5.6 | 79.4 | | |
| No change | 2 | 1.8[a] | 23.2[a] | 7.5 | 135.8 |
| | 12 | 7.2 | 143.6 | 12.2 | 179.3 |
| | 15 | 9.0 | 107.8 | 15.8 | 201.7 |
| | 17 | 9.5 | 125.5 | 14.9 | 297.6 |
| | 18 | 10.3 | 183.2 | 10.2 | 156.1 |
| | 19 | 11.0 | 137.8 | 17.2 | 308.0 |
| | Median | 9.3 | 131.6 | 12.8 | 190.9 |
| Partial response | 3 | 2.9[a] | 56.2[a] | 9.9 | 139.5 |
| | 5 | 17.4 | 188.4 | 17.6 | 203.0 |
| | 6 | 7.7 | 118.3 | | |
| | 8 | 5.9 | 118.7 | 10.0 | 175.2 |
| | 10 | 14.8 | 206.6 | 9.1 | 191.7 |
| | 14 | 9.1 | 134.6 | 13.5 | 266.0 |
| | 16 | 4.4 | 72.8 | 12.0 | 190.8 |
| | Median | 7.7 | 118.7 | 11.0 | 191.0 |

[a] Patients 1–4 received 100 mg dose at entry and 250 mg dose from month 2 onwards.

**Figure 3** Median and interquartile ranges of lipids and hormones during treatment with ICI 182780. Numbers above the curves refer to the numbers of patients tested. Twelve patients were tested for the four lipids (CHOL, cholesterol; TRIG, triglyceride; HDLC, high-density lipoprotein; LDLC, low-density lipoprotein), 19 for the LH, FSH and SHBG and 13 for prolactin. Numbers decline because patients go off study after progression.

**Figure 4** Serial ultrasound estimations of endometrial thickness in five patients. Thickened endometrium compared with normal post-menopausal women was thought to be due to treatment with tamoxifen. No significant change occurred up to 15 months of treatment with ICI 182780.

formed in five responding patients. Endometrial thickness was greater than the expected <2 mm usually found in post-menopausal women, in all patients. The thickness of the endometrium remained unchanged in all patients during treatment with ICI 182780 (Figure 4). Endometrial histology was reviewed on one patient who had a hysterectomy. This was reported as showing an atrophic post-menopausal pattern with cystic change. The glands were lined by flattened and cuboidal epithelium. There was no mitotic activity, epithelial ectoplasia or polyp formation. There were

**Table III** Response rate and durations of response to ICI 182780 in relation to duration of previous treatment with tamoxifen

| *Response ICI 182780* | *Number (%)* | *Durations (months)* |
|---|---|---|
| Partial | 7 (37) | 25+ (PR19)[a], 33+ (NC8), 32+ (PR24) 25+ (A67), 12 (A74), 8 (A20), 3 (A77) |
| No change | 6 (32) | 29+ (NC8), 33+ (A74), 23+ (A61), 30+ (NC48), 30+ (A23), 9 (NC34) |
| Progression | 6 (31) | All patients progressed in <8 weeks (A48, A45, NC7, PR8, A9, NC12) |

[a]Letters in brackets indicate response to tamoxifen when given for advanced disease (PR, partial remission; NC, no change; PD, progressive disease) or if given as an adjuvant therapy (A). The numbers in the brackets indicate duration of treatment with tamoxifen.

no significant changes in body weight during treatment with ICI 182870. Mean body weight (kg$\pm$s.d.) was $63.8\pm14.0$ (n = 13) at the beginning of treatment, $64.9\pm15.8$ ($n$ = 11) after 6 months, $64.5\pm17.2$ ($n$ = 9) after 10 months and $64.2\pm18.3$ ($n$ = 9) after 16 months of treatment with ICI 182780.

*Response*

All 19 patients are evaluable for response to ICI 182780 (Table III). Six patients were unresponsive *de novo* and showed objective evidence of disease progression within 2 months of commencing treatment. The remaining 13 patients (69%) all responded to treatment with the specific anti-oestrogen for a median duration of 25 months. Seven patients (37%) showed PRs for 33+, 32+, 25+, 25, 12, 8 and 3 months, and six patients (32%) showed NC responses for 33+, 30+, 30+, 29, 23 and 9 months. Thus five patients are still in remission and continuing treatment with ICI 182780 after 30–33 months. Responses have been observed in six of the nine women who progressed while receiving tamoxifen as treatment for advanced breast cancer as well as in seven of the ten women who relapsed after treatment with tamoxifen as adjuvant therapy. There appeared to be no association between duration of treatment with tamoxifen and subsequent response to ICI 182780 (Table III).

### Discussion

This study represents the first investigation of long-term administration of the specific anti-oestrogen, ICI 182780, to patients with breast cancer and demonstrates that predicted therapeutic levels of ICI 182780, as judged from animal experiments (Wakeling *et al.*, 1991; Dukes *et al.*, 1993) and our previous short phase I study (DeFriend *et al.*, 1994*b*) can be achieved and maintained for 1 month following a single i.m. injection of the long-acting formulation used. Treatment with ICI 182780 was associated with minor effects on serum hormones and lipid levels, produced few side-effects and resulted in a high response rate after tamoxifen failure, together with a median reponse duration of 25 months.

Pharmacokinetic data concerning the release characteristics of the drug into the serum in this study were found to be similar to those previously demonstrated in adult female monkeys (Dukes *et al.*, 1993). From studies on inhibition of endometrial proliferation in the monkey and inhibition of tumour proliferation in a previous phase I study, it was predicted that serum levels of ICI 182780 in the range of 2–3 ng $ml^{-1}$ were consistent with a therapeutic effect in patients with advanced breast cancer. However, a direct pharmacokinetic–pharmacodynamic link is not proven with the few patients studied to date. Serum drug concentrations in excess of this were observed with the 250 mg dose used in the present study for most of the first and all of the sixth month. However, there was evidence of drug accumulation after multiple dosing, such that after 6 months treatment there was an 80% increase in mean end of month drug levels and a 50% increase in the AUC compared with data from month 1. These data suggest that lower doses of the drug may be effective in maintaining therapeutic serum drug levels, although further clinical studies are required to confirm this hypothesis.

Previous animal studies with ICI 182780 have shown that the activity of specific anti-oestrogens may vary between different oestrogen target sites (Wakeling, 1993). In the present study, we have attempted to obtain preliminary data on the effects of long-term administration of ICI 182780 on the pituitary gland/hypothalamus, the liver and the endometrium. Serum gonadotrophin levels significantly increased during the first 3 months of treatment with ICI 182780 and then remained stable thereafter. This change is in contradistinction to that seen with the triphenylethylene anti-oestrogen, tamoxifen, which reduces serum levels of FSH and LH in post-menopausal patients because of its oestrogenic effect on the pituitary gland and hypothalamus (Willis *et al.*, 1977). All but four patients in the present study were treated with tamoxifen up until treatment with ICI 182780 was initiated. Levels of FSH and LH at this time were below the range for normal post-menopausal women, which is attributable to previous therapy with tamoxifen. The rise of gonadotrophins during the first 3 months of treatment with ICI 182780 may therefore have been a passive effect caused by cessation of tamoxifen rather than an active anti-oestrogen effect of the new agent. The fact that gonadotrophin levels rose to well within post-menopausal values and then remained stable would support the view that ICI 182780 is without effect on gonadotrophin levels. This apparent lack of activity of the specific anti-oestrogen on the hypothalamus and pituitary gland is further supported by the findings that ICI 182780 did not initiate or exacerbate hot flushes or sweats in the present study, nor did it produce significant changes in serum prolactin levels.

Treatment with tamoxifen has been reported to increase serum levels of SHBG secondary to a probable oestrogenic effect of tamoxifen on the liver (Sakai *et al.*, 1978). The levels of SHBG in some of our patients before starting therapy with ICI 182780 were high, consistent with the oestrogenic effect of prior treatment with tamoxifen. Following commencement of treatment with ICI 182780, a slight decline in SHBG levels occurred, consistent with tamoxifen withdrawal, but thereafter median levels of SHBG remained in the middle of the normal range for our laboratory, suggesting that the specific anti-oestrogen may have little effect on SHBG synthesis in the liver. A lack of effect of the compound on the liver is further suggested by evaluation of lipid changes. Tamoxifen is known to reduce levels of cholesterol and LDL cholesterol and is associated with an increase in triglycerides and HDL cholesterol consistent with an oestrogenic effect on the liver (Bertelli *et al.*, 1988; Love *et al.* 1990). None of these changes reported during treatment with tamoxifen were observed during the present study. This would further suggest that ICI 182780 is peripherally selective with respect to the liver. However we cannot explain why there was not the expected changes in lipids as patients came off tamoxifen.

The lack of apparent adverse effects of ICI 182780 seen in the present study would, if confirmed in future larger trials, give the specific anti-oestrogen potential advantages over currently available second-line endocrine agents. The observed lack of effect of ICI 182780 on body weight over a period of study that ranged from 6 to 16 months would give the new agent a potential advantage over megestrol acetate, the most widely used second-line endocrine therapy in breast cancer, which resulted in weight gain of >5% of body weight in 25% of patients in one study (Willemse *et al.*, 1990) and a median weight gain of 9 lbs after 180 days of treatment in another (Cruz *et al.*, 1990). Overall, 83% of patients reported side-effects during treatment with megestrol acetate (Willemse *et al.*, 1990).

The most troublesome side-effects of tamoxifen, the current first-line endocrine therapy of choice, are the inception or exacerbation of hot flushes and sweats and the initiation of vaginal discharge. As already stated, ICI 182780 did not induce or exacerbate hot flushes or sweats in the present study and furthermore did not cause symptoms of vaginal dryness or altered libido. Since the vaginal discharge experienced by 10–33% of patients during tamoxifen therapy is thought to be related to the oestrogenic activity of the drug, we expected ICI 182780 to be associated with vaginal dryness and irritation; the fact that this did not occur further suggests that ICI 182780 may be peripherally selective with respect to oestrogen target-site activity.

Serial measurements of endometrial thickness were obtained from five patients during treatment with ICI 182780. Before commencing treatment with the specific anti-oestrogen, the endometrial thickness in all five patients was greater than that found in the normal post-menopausal uterus. We assume that the overall 'thickening' we detected was related to previous tamoxifen therapy as this phenomenon has been widely reported. During the relatively short duration of the present study, there was no further increase in thickening during treatment with ICI 182780. However there was also no decline in thickness as might be expected following treatment with a specific anti-oestrogen. Whether this finding reflected a true increase in endometrial thickness or an additional swelling of the myometrium, which has also been described in women taking tamoxifen (Cohen *et al.*, 1994), is not known as we did not perform endometrial biopsies. In the one patient where hysterectomy was performed, histology showed no thickening of the endometrium, rather that it was flattened, atrophic and with no mitotic activity. From the results of the sequential ultrasound data and the endometrial histology, it is not clear why ICI 182780 does not result in thinning of the endometrium. In primates (Dukes *et al.*, 1993) and in short-term studies in women (Thomas *et al.*, 1993) ICI 182780 inhibited endometrial proliferation at similar serum concentrations to those seen in the present study. It is possible, therefore, that

ICI 182780 inhibits further endometrial proliferation but does not cause regression of pre-existing hypertrophied endometrial tissue. If a similar inhibitory effect of ICI 182780 on endometrial proliferation is proven in future longer term clinical studies, it would suggest a further potential therapeutic advantage of specific anti-oestrogens over partial agonist agents, as the oestrogenic activity of tamoxifen on the female genital tract has led to concerns over its potential to induce endometrial hyperplasia or carcinoma (Fornander *et al.*, 1989) in patients receiving adjuvant therapy or entering breast cancer prevention trials.

The response data in the present study show that patients with advanced breast cancer have a high chance of response for prolonged durations to a specific anti-oestrogen after failure of treatment with a partial agonist anti-oestrogen. In the highly selected group of patients reported here, there appeared to be no cross-resistence between ICI 182780 and taxomifen in 69% of patients and the median duration of reponse was 25 months. In contrast, clinical studies in which tamoxifen-resistant patients were treated with another triphenylethylene anti-oestrogen, toremifene, demonstrated much higher rates of cross-resistance (Vogel *et al.*, 1993 and references therein).

ICI 182780 is thought to act exclusively as an anti-oestrogen via the ER (Wakeling *et al.*, 1991). Responses to treatment with ICI 182780 after progression on tamoxifen in such patients suggests that tamoxifen failure may be because of the intrinsic agonist activity of tamoxifen or one of its oestrogenic metabolites (Simon *et al.*, 1984; Gottardis *et al.*, 1989; Osborne *et al.*, 1991; Howell *et al.*, 1992; DeFriend *et al.*, 1994*a*) or because tamoxifen is, in some way, sequestered from the ER (Pavlick *et al.*, 1992; Wolfe *et al.*, 1993) allowing endogenous oestradiol to recommence tumour stimulation.

Tamoxifen has been shown to stimulate the growth of human mammary tumour cell lines when grown both *in vitro* (Lykkesfeldt and Sorenson, 1992) and *in vivo* as xenografts in nude mice (Gottardis *et al.*, 1989). We have reported preliminary data demonstrating tamoxifen-induced growth stimulation *in vitro* of breast cancer cells harvested from patients with advanced disease at the time of failure of tamoxifen treatment (DeFriend *et al.*, 1994*a*). In all the experiments cited above, the specific anti-oestrogen was able to reverse the stimulatory effects of tamoxifen. In addition, we and others have demonstrated so-called withdrawal responses in breast cancer patients after stopping treatment with tamoxifen at the time of tumour progression, further suggesting tumour stimulation by tamoxifen as a possible cause of treatment failure (Howell *et al.*, 1992). However, in most studies withdrawal responses occur in only one-third or less of patients and thus tamoxifen withdrawal responses are unlikely to account for all the responses seen after treatment with ICI 182780 in the current study.

Alternatively, there is also evidence that tamoxifen may be either excluded from tumour cells (Osborne *et al.*, 1991) or possibly sequestered away from the ER at alternative intracellular binding sites (Pavlick *et al.*, 1992), allowing endogenous oestrogens to restimulate tumour growth and cause treatment failure. Under these circumstances tumours would be expected to retain responsiveness to a specific anti-oestrogen and in *in vitro* studies, human mammary tumour cell lines that are unresponsive to tamoxifen have been shown to retain sensitivity to the antiproliferative effects of ICI 182780 (Brunner *et al.*, Lykkesfeldt *et al.*, 1994; Coopman *et al.*, 1994).

As neither of the two possible mechanisms of tamoxifen resistance outlined above (tamoxifen agonism or tamoxifen sequestration) would be expected to be shared by the chemically distinct, specific anti-oestrogens, the new agents have the potential to improve the rate and/or duration of response to anti-oestrogen therapy in breast cancer. Furthermore, there is experimental evidence to suggest that specific anti-oestrogens may also prove to be more effective than alternative strategies for oestrogen deprivation of human breast tumours, including the use of gonadotrophin-releasing hormone analogues and aromatase inhibitors.

These alternative treatments profoundly reduce but do not completely abolish oestrogen synthesis and small amounts of circulating oestrogen remain detectable in the serum of patients. Although these very low levels of oestrogen may be insufficient to produce any significant directly mitogenic effects in breast cancer cells, recent experimental studies have shown that small concentrations of oestradiol can act synergistically to amplify the effects of other growth-promoting pathways such as epidermal growth factor/transforming growth factor alpha (EGF/TGF-$\alpha$), insulin-like growth factor (IGF)-1 and the fibroblast growth factor family (Stewart *et al.*, 1990; Westley and May, 1991). In addition, EGF and dopamine have been found to activate ER signalling pathways in the complete absence of ligand and it has been shown that an EGF-specific tyrosine kinase inhibitor can inhibit both proliferation and expression of several oestrogen-responsive genes by MCF-7 cells under conditions of complete oestrogen withdrawal (Reddy *et al.*, 1992; Wakeling *et al.*, 1994).

Specific anti-oestrogens produce both potent inhibition of ER activation by oestrogen and reduction of ER levels. In *in vivo* studies, they have been found to reduce the growth rate of breast cancer cells, grown in culture media containing no exogenous oestrogens, and to inhibit activation of ER signalling pathways by EGF and dopamine (Power *et al.*, 1991; Ignar-Trowbridge *et al.*, 1992). Specific anti-oestrogens may therefore be superior to other methods of oestrogen withdrawal for the treatment of breast cancer, as they appear to prevent activation of growth pathways by remaining small amounts of oestrogen and also to block constitutive ER activation, stimulated by non-oestrogen-mediated pathways.

We conclude from the results of this preliminary study that the pure anti-oestrogen, ICI 182780, is well tolerated during long-term treatment and is active as an anti-tumour agent in patients with advanced breast cancer who have previously relapsed on tamoxifen. At the dose used, there was accumulation of the drug over time and thus lower doses than those administered in this study may be as effective. The evident lack of cross-resistance between tamoxifen and ICI 182780 is in accordance with the hypothesis that the agonist activity or sequestration of tamoxifen may cause some treatment failures in patients with advanced breast cancer and results in restimulation of tumour growth. Since ICI 182780 appears devoid of agonist activity, treatment failure via a similar mechanism should not occur, and it is possible, therefore, that this new agent may improve the rate and duration of response in patients with advanced breast cancer. However, further studies are required to confirm the response rate and also to determine the long-term effects of this agent on bone, plasma lipids and the endometrium.

**Acknowledgements**

We thank Sisters Julie Kiernan and Nikki Scott for services in the clinical study, Messrs E Hoare, JMT Howat and RJ Williams for referring patients, Dr M Anderson, Reader in Pathology, Queens Medical Centre, Nottingham, for reviewing the endometrial histology, Jean Miller for help with preparing the manuscript, Linda Ashcroft for statistical advice and Zeneca Pharmaceuticals for providing financial support.

### References

BERTELLI G, PRONZATO P, AMOROSO D, CUSIMANO MP, CONTE PF, MONTAGNA G, BERTOLONI S AND ROSSO R. (1988). Adjuvant tamoxifen in primary breast cancer: influence on plasma lipids and antithrombin III levels. *Breast Cancer Res. Treat.*, **12**, 307–310.

BRUNNER N, BOYSON B, KILGAARD JF, JIRUS S AND CLARKE R. (1993*a*). Resistance to 4OH-tamoxifen does not confer resistance to the steroidal antioestrogen ICI 182780, while acquired resistance to ICI 182780 results in cross-resistance to 4OH-tamoxifen. (abstract 19). *Breast Cancer Res. Treat.*, **27**, 135.

BRUNNER N, FRANDSEN TL, HOLST-HANSEN C, BEI M, THOMPSON EW, WAKELING AE, LIPPMAN ME AND CLARKE R. (1993*b*). MCF-7/LCC2: a 4-hydroxy-tamoxifen resistant human breast cancer variant which retains sensitivity to the steroidal antioestrogen ICI 182780. *Cancer Res.*, **53**, 3229–3232.

CLARKE RB, LAIDLAW IJ, JONES LJ, HOWELL A AND ANDERSON E. (1993). Effect of tamoxifen on Ki67 labelling index in human breast tumours and its relationship to oestrogen and progesterone receptor status. *Br. J. Cancer*, **67**, 606–611.

COHEN I, ROSEN DJ, TEPPER R, CORDOBA M, SHAPIRA Y, ALTARAS MM, YIGAEL D AND BEYTH Y. (1994). Ultrasonographic evaluation of the endometrium and correlation with endometrial sampling in postmenopausal patients treated with tamoxifen. *J. Ultrasound Med.*, **12**, 275–280.

COLE MP, JONES CTA AND TODD IDJ. (1971). A new antioestrogenic agent in late breast cancer. An early clinical appraisal of ICI 46,474. *Br. J. Cancer*, **25**, 270–275.

COOPMAN P, GARCIA M, BRUNNER N, DEROCQ D, CLARKE R AND ROCHEFORT H. (1994). Anti-proliferative and anti-oestrogenic effects of ICI 164,384 and ICI 182,780 in 4-OH-Tamoxifen resistant human breast cancer cells. *Int. J. Cancer*, **56**, 295–300.

CRUZ JM, MUSS HB, BROCKSCHMIDT JK AND EVANS GW. (1990). Weight changes in women with metastatic breast cancer treated with megestrol acetate: a comparison of standard versus high-dose therapy. *Semin. Oncol.*, **17**, 63–67.

DAUVOIS S, DANIELIAN PS, WHITE R AND PARKER MG. (1992). Antioestrogen ICI 164384 reduced cellular estrogen receptor content by increasing its turnover. *Proc. Natl. Acad. Sci. USA*, **89**, 4037–4041.

DEFRIEND DJ, ANDERSON E, BELL J, WILKS DP, WEST CML AND HOWELL A. (1994*a*). Effects of 4-hydroxytamoxifen and a pure antioestrogen (ICI 182780) on the clonogenic growth of human breast cancer cells in vitro. *Br. J. Cancer*, **70**, 2043–211.

DEFRIEND DJ, HOWELL A, NICHOLSON RI, ANDERSON E, DOWSETT M, MANSEL RE, BLAMEY RW, BUNDRED NJ, ROBERTSON JF, SAUNDERS C, BAUM M, WALTON P, SUTCLIFFE F AND WAKELING AE. (1994*b*). Investigation of a new pure antiestrogen (ICI 182780) in women with primary breast cancer. *Cancer Res.*, **54**, 408–414.

DUKES M, WATERTON JC, WAKELING AE. (1993). Antiuterotrophic effects of the pure antioestrogen ICI 182,780 in adult female monkeys (*Macaca-nemestrina*)–Quantitative magnetic resonance imaging. *J. Endocr.*, **138**, 203.

FAWELL SE, WHITE R, HOARE S, SYDENHAM M, PAGE M AND PARKER MG. (1990). Inhibition of oestrogen receptor-DNA binding by the 'pure' antioestrogen ICI 164,384 appears to be mediated by impaired receptor dimerization. *Proc. Natl Acad. Sci. USA*, **87**, 6883–6887.

FORNANDER T, RUTQVIST LE AND CEDERMARK B. (1989). Adjuvant tamoxifen in early breast cancer: occurrence of new primary cancers. *Lancet*, **1**, 117–120.

FRIEDEWALD WT, LEVI RI AND FREDRICKSON DS. (1972). Estimation of the concentration of low-density lipoprotein cholesterol in plasma, without use of the preparative ultracentrifuge. *Clin. Chem.*, **18**, 499–502.

GOTTARDIS MM, JIANG SY, YENG MH, JORDAN VC. (1989). Inhibition of tamoxifen-stimulated growth of an MCF-7 variant in athymic mice by novel steroidal antioestrogens. *Cancer Res.*, **49**, 4090–4093.

HAYWARD JL, CARBONE PP, HEUSON JC, KUMASKA S, SEGALOFF A AND RUBENS RD. (1977). Assessment of response to therapy in advanced breast cancer. *Eur. J. Cancer*, **13**, 11–33.

HOWELL A, MACKINTOSH J, JONES M, REDFORD J, WAGSTAFF J AND SELLWOOD RA. (1988). The definition of the 'No Change' category in patients treated with endocrine therapy and chemotherapy for advanced carcinoma of the breast. *Eur. J. Cancer Clin. Oncol.*, **24**, 156–157.

HOWELL A, DODWELL D, LAIDLAW I, ANDERSON H AND ANDERSON E. (1990). Tamoxifen as an agonist for metastatic breast cancer. In *Endocrine Therapy of Breast Cancer* IV, Goldhirsch A (ed.) pp. 49–58. Springer: Berlin.

HOWELL A, DODWELL D, ANDERSON H AND REDFORD J. (1992). Response after withdrawal of tamoxifen and progestogens in advanced breast cancer. *Ann. Oncol.*, **3**, 611–617.

HOWELL A, DE FRIEND D, ROBERTSON J, BLAMEY R AND WALTON P. (1995). Response to a specific antioestrogen (ICI 182780) in tamoxifen-resistant breast cancer. *Lancet*, **345**, 29–30.

IGNAR-TROWBRIDGE DM, NELSON KG, BIDWELL MC, CURTIS SW, WASHBURN TF, MC LACHLAN JA AND KORACH KS. (1992). Coupling of dual signalling pathways: epidermal growth factor action involves the oestrogen receptor. *Proc. Natl Acad. Sci. USA*, **89**, 4658–4662.

LOVE R, NEWCOMB P AND WIEBE D. (1990). Effects of tamoxifen therapy on lipid and lipoprotein levels in postmenopausal patients with node-negative breast cancer. *J. Natl Cancer Inst.*, **82**, 1327–1332.

LYKKESFELDT AE AND SORENSEN EK. (1992). Effect of oestrogen and anti-oestrogen on cell-proliferation and synthesis of secreted proteins in the human breast cancer cell line MCF-7 and a tamoxifen resistant variant subline, AL-1. *Acta Oncol.*, **31**, 131–138.

LYKKESFELDT AE, MADSEN MW AND BRIAND P. (1994). Altered expression of estrogen-regulated genes in a tamoxifen-resistant and ICI 164,384 and ICI 182,780 sensitive human breast cancer cell line, MCF-7/TAM$^{R}$-1$^{1}$. *Cancer Res*, **54**, 1587–1595.

NICHOLSON RI, FRANCIS AB, MCCLELLAND RA, MANNING DL AND GEE JMW. (1994). Pure anti-oestrogens (ICI 164384 and ICI 182780) and breast cancer: is the attainment of complete oestrogen withdrawal worthwhile? *Endocrine Related Cancer*, **1**, 5–17.

OSBORNE CK, CORONADO E AND ALLRED CD. (1991). Acquired tamoxifen resistance: correlation with reduced breast tumor levels of tamoxifen and isomerization of trans-4-hydroxytamoxifen. *J. Natl Cancer Inst.*, **83**, 1477–1482.

PATTERSON JS, EDWARDS DG AND BATTERSBY LA. (1981). A review of the international clinical experience with tamoxifen. *Jpn J. Cancer Clin.*, Supplement (November), 157–183.

PAVLIK EJ, NELSON K AND SRINIVASAN S. (1992). Resistance to tamoxifen with persisting sensitivity to estrogen: possible mediation by excessive antiestrogen binding site activity. *Cancer Res.*, **52**, 4106–4112.

POWER RF, MANI SK, CODINA J, CONNEELLY OM AND O'MALLEY BW. (1991). Dopaminergic and ligand-independent activation of steroid hormone receptors. *Science*, **244**, 1636–1639.

REDDY KB, MANGOLD GL, TANDON AK, YONEDA T, MUNDY GR, ZILBERSTEIN A AND OSBORNE CK. (1992). Inhibition of breast cancer cell growth in vitro by a tyrosine kinase inhibitor. *Cancer Res.*, **52**, 3636–3641.

ROBERTSTON JFR, WILLIAMS MR, TODD J, NICHOLSON RI, MORGAN DAL AND BLAMEY RW. (1989). Factors predicting the response of patients with advanced breast cancer to endocrine (Megace) therapy. *Eur. J. Cancer. Clin. Oncol.*, **25**, 469–475.

ROBERTSON JFR, ELLIS IO, NICHOLSON RI, ROBINS A, BELL J AND BLAMEY RW. (1991). Cellular effects of tamoxifen in primary breast cancer. *Breast Cancer Res. Treat.*, **20**, 117–123.

SAKAI F, CHEIX F, CLAVEL M, COLON J, MAYER M, PANNATA F AND SAEZ S. (1978). Increases in steroid binding globulins induced by tamoxifen in patients with carcinoma of the breast. *J. Endocrinology*, **76**, 219–226.

SIMON W, ALBRECHT M AND TRAMS G. (1984). In-vitro growth promotion of human mammary carcinoma cells by steroidal hormones, tamoxifen and prolactin. *J. Natl Cancer Inst.*, **73**, 313–321.

STEWART AJ, WESTLEY BR, MAY FEB AND WESTLEY BR. (1990). Modulation of proliferative response of breast cancer cells to growth factors by oestrogen. *Br. J. Cancer*, **66**, 640–648.

THOMAS EJ, THOMAS NM, WALTON PL AND DOWSETT M. (1993). The effects of ICI 182,780, a pure antioestrogen on reproductive endocrinology in normal pre-menopausal women. *J. Endocrinol.*, **137S**, 183.

TURKEN S, SIRIS E AND SELDON. (1989). Effects of tamoxifen on spinal bone density in women with breast cancer. *J. Natl Cancer Inst.*, **81**, 1086–1088.

VOGEL CL, SHEMANO I, SCHOENFELDER J, GAMS RA AND GREEN MR. (1993). Multicenter phase II efficacy trial of toremifene in tamoxifen-refractory patients with advanced breast cancer. *J. Clin. Oncol.*, **11**, 345–350.

WAKELING AE. (1993). The future of new pure antiestrogens in clinical breast cancer. *Breast Cancer Res. Treat.*, **25**, 1–9.

WAKELING AE AND BOWLER J. (1987). Steroidal pure anti-oestrogens. *J. Endocrinol.*, **112**, R7–R10.

WAKELING AE AND BOWLER J. (1988). Novel anti-oestrogens without partial agonist activity. *J. Steroid Biochem.*, **31**, 645–653.

WAKELING AE, NEWBOULT E AND PETERS SW. (1989). Effects of anti-oestrogens on the proliferation of MCF-7 human breast cancer cells. *J. Mol. Endocrinol.*, **2**, 225–234.

WAKELING AE, DUKES M AND BOWLER J. (1991). A potent specific pure antioestrogen with clinical potential. *Cancer Res.*, **51**, 3867–3873.

WAKELING AE, BARKER AJ, DAVIES DH, BROWN DS, GREEN LR, CARTLIDGE SA AND WOODBURN JR. (1994). Inhibition of EGF-receptor tyrosine kinase activity by 4-aniloquinazolines. (abstract 6.6). *Br. J. Cancer*, **69** (suppl. 21), 18.

WESTLEY BR AND MAY FEB. (1991). IGF's and control of cell proliferation in breast and other cancers. *Rev. Endocrine Rel. Cancer*, **39**, 29–34.

WILLEMSE PHB, VAN DER PLOEG E, SLEIJFER D, TJABBES T AND VAN VEELEN HA. (1990). A randomized comparison of megestrol acetate (MA) and medroxyprogesterone acetate (MPA) in patients with advanced breast cancer. *Eur. J. Cancer*, **26**, 337–343.

WILLIS KJ, LONDON DR, WARD HWC, BUTT WR, LYNCH SS AND RUDD BT. (1977). Recurrent breast cancer treated with the antioestrogen tamoxifen: correlation between hormonal changes and clinical course. *Br. Med. J.*, **1**, 425–428.

WOLF DM, LANGAN-FAHEY, PARKER CJ, MCGAGUE R AND CRAIG JORDAN V. (1993). Investigation of the mechanism of tamoxifen-stimulated breast tumour growth with nonisomerizable analogues of tamoxifen and metabolites. *J. Natl Cancer Inst.*, **85**, 806–812.